# A multidisciplinary team nursing model in the treatment of patients undergoing transapical mitral valve clamping: a prospective study

**DOI:** 10.1186/s13019-021-01588-7

**Published:** 2021-07-28

**Authors:** Sailan Li, Haoruo Zhang, Meihua Chen, Zhenzhen Wang, Yanjuan Lin

**Affiliations:** 1 Department of Cardiac Surgery, Fujian Medical University Union Hospital, 29 Xinquan Road, Fuzhou City, Fujian Province China; 2 Fujian Medical University Union Hospital, 29 Xinquan Road, Fuzhou City, Fujian Province China; 3 Department of Nursing, Fujian Medical University Union Hospital, 29 Xinquan Road, Fuzhou City, Fujian Province China

**Keywords:** Cardiac insufficiency, Transapical mitral valve clamp surgery, Multidisciplinary team, Nurses, soins multidisciplinaires, pinchage de la valve mitral, chirurgie cardiaque

## Abstract

**Background:**

As a new surgical method for older adults with cardiac insufficiency, transapical mitral valve clamp surgery requires the cooperation of practitioners across multiple disciplines to ensure appropriate treatment and nursing care. This study aimed to explore the utility of a multidisciplinary team nursing model in the clinical treatment and nursing care of patients undergoing transapical mitral valve clamping.

**Methods:**

Our sample of ten patients included four men (40%) and six women (60%), with a mean age of 71.4 ± 5.2 years. The multidisciplinary team comprised nurses that specialized in severe illness, cardiac health, rehabilitation, psychology, nutrition, and pain. The team engaged in comprehensive discussions regarding problems specific to the patients undergoing transapical mitral valve surgery, allowing them to formulate individualized nursing measures and implement precise policies.

**Results:**

No serious postoperative complications occurred in any of the ten patients included in this study, and a significant improvement was noted in the cardiac status of all the patients. Color ultrasound findings at discharge indicated that the degree of reflux of all the patients was ≤2+. Among the ten patients, the Activity of Daily Living Scale scores at discharge were significantly higher than before the operation (69.0 ± 4.6 vs. 55.0 ± 5.8). In addition, the 6-min walking test results at discharge were significantly better than those observed before the operation (318.0 ± 21.7 m vs. 295.2 ± 18.4 m).

**Conclusions:**

Utilization of a multidisciplinary team allows nurses across various specialties to provide more comprehensive and systematic care for patients undergoing a mitral valve clamping operation, thus promoting patient recovery.

## Résumé

**Contexte:**

en tant que nouvelle méthode chirurgicale pour le traitement de l’insuffisance cardiaque chez les personnes âgées, la chirurgie par pince de la valve mitral aiguë nécessite la collaboration de médecins multidisciplinaires pour assurer un traitement et des soins appropriés.

**Objectifs:**

étudier l’application du modèle de soins en équipe multidisciplinaire dans le traitement clinique et les soins aux patients atteints de la valve mitral par pince.

**Méthode:**

sur 10 patients, 4 hommes (40%) et 6 femmes (60%) ont un âge moyen de 71.4 à 5.2 ans. L’équipe multidisciplinaire est. composée d’infirmiers spécialisés dans les maladies graves, la santé cardiaque, la réadaptation, la psychologie, la nutrition et la douleur.

L’équipe a mené une discussion approfondie sur les problèmes spécifiques des patients soumis à une opération de la valve mitral transspiroptérale, a élaboré des mesures de soins personnalisées et a mis en œuvre des politiques précises.

**Résultats:**

aucune complication postoperatoire grave n’a été observée chez les 10 patients de ce groupe, et les défaillances cardiaques ont été nettement améliorées chez tous les patients. Une double échographie en couleur a montré que le degré de regurgitation mitral était inférieur ou égal à 2+ dans 10 patients sortant de cette étude; Les scores sur l’échelle des activités de la vie quotidienne à la sortie de l’hôpital étaient nettement plus élevés dans 10 cas que dans le cas préopératoire (69.0 ± 4.6 contre 55.0 ± 5.8). En outre, les résultats d’un essai de marche de 6 min à la sortie de l’hôpital étaient nettement supérieurs à ceux observés avant l’opération (318.0 ± 21.7 m contre 295.2 ± 18.4 m).

**Conclusion:**

le recours à des équipes multidisciplinaires permet aux infirmiers/infirmières spécialisés de fournir des soins plus complets et systématiques aux patients soumis à la chirurgie par compression de la valve mitral, ce qui facilite leur réadaptation.

## Background

Mitral regurgitation is a common valvular heart disease, with incidence rates of 6.4% in people over 65 years of age and 9.3% in those over 75 years of age [1]. Studies have confirmed that patients with moderate to severe mitral regurgitation, exhibiting cardiac insufficiency, have a poor prognosis [2]. Valve replacement or repair under surgical thoracotomy is the first-line choice for patients with mitral regurgitation. However, in patients with advanced age, histories of thoracotomy, poor cardiac functions, and multiple organ dysfunctions, it is not an effective choice because they cannot tolerate surgery [3]. In these patients, transapical mitral valve clamping is more appropriate, as it is associated with less trauma [4]. During the procedure, the mitral ValveClamp (Hanyu Medical Technology, Shanghai, China) is used to clamp the middle of the anterior and posterior lobes of the mitral valve, such that the larger outlet of the systolic mitral valve becomes two smaller outlets, thereby reducing valve regurgitation. The principle of this technology is similar to that of the MitraClip, which is currently used worldwide. However, when compared with the MitraClip, the ValveClamp has the advantages of direct puncture from the heart tip, a short implantation path, avoidance of vascular injuries, and an independence from peripheral vascular conditions. The appropriate positioning of the implanted clamp can be observed using esophageal ultrasound. In addition to utilizing smaller delivery system models, the ValveClamp offers the advantages of a larger capture range, wider indications, and simpler operation than the MitraClip [5, 6].

Multidisciplinary teams (MDTs) are utilized in the treatment of certain clinical diseases, allowing for the formulation of the most reasonable, standardized, individualized, and continuously comprehensive treatment plans by means of multidisciplinary discussions [7]. In 2014, experts from the American Heart Association, the American College of Cardiology, and experts from organizations such as the American Society of Thoracic Surgeons completed and released, jointly, the 2014 Guidelines and Executive Summary for the Management of Patients with Heart Valve Disease. The guide emphasized that a comprehensive MDT is the basis of transcatheter valve treatment of patients with heart valve disease [8]. As a new surgical method for older adults with cardiac insufficiency, transapical mitral valve clamp surgery requires the cooperation of practitioners across multiple disciplines to ensure appropriate treatment and nursing care. Therefore, to improve cure rates of transapical mitral valve clamping patients, we investigated the effects of greater systematic and comprehensive discussion using rapid rehabilitation nursing with the involvement of MDTs.

## Materials and methods

### Participants

Patients undergoing transapical mitral valve clamping aged > 18 years admitted to the Heart Medical Centre of the Fujian Medical University Union Hospital between June 2020 and June 2021 were included in this prospective study. Patients with severe mental illnesses were excluded. The study was conducted according to the guidelines laid down in the Declaration of Helsinki, and all procedures involving human participants/patients were approved by the Ethics Committee of the Fujian Medical University Union Hospital (ethics number, 2019YX010–01). Written informed consent was obtained from all participants.

### Surgical methods

The patient was placed in the surgical position. After routine disinfection and general anesthesia, the apex of the fifth intercostal heart was cut and separated, the heart apex exposed, the purse string sutured, and then the apex punctured. With the aid of transesophageal echocardiography, the arms of the clamp were placed perpendicular to the opening line of the mitral valve. After capturing the mitral valve leaflets, the clamp was tightened, the delivery system withdrawn from the body, and the mitral valve clamp implanted. The degree of mitral regurgitation was re-examined by transesophageal echocardiography. The apical wound was sutured to close the chest cavity. Anesthesia was discontinued, and the patient was transferred to the intensive care unit (ICU) for intensive care and symptomatic treatment.

### Establishing a multidisciplinary collaborative nursing team

The head nurse of the Cardiac Surgery ICU served as the team leader, and the team members covered multiple disciplines such as critical illness, cardiac health, rehabilitation, psychology, nutrition, and pain, among others. The group held regular meetings to formulate work procedures, cooperation methods and systems, and established a WeChat group to strengthen daily work communication. To protect the privacy of patients, the research objects were numbered, and the work plan was discussed in the daily work with the number. The routine process for multidisciplinary nursing was as follows. After the morning meeting and shift change in the department, multidisciplinary nursing rounds were arranged, following which individualized nursing plans were negotiated jointly, and the responsible nurses were required to carry out daily nursing work in accordance with the plan. In the afternoon, the responsible nurse conducted rounds again, evaluated the completion of the nursing plan, and reported the status of completion and existing problems on the WeChat group. The team leader summarized the findings according to the reported situation, discussed and improved the plan for the next set of rounds or meetings, and formulated a high-quality management model for sustainable quality improvement.

### Work contents of multidisciplinary cooperative nursing group

#### Preoperative nursing

##### Mental preparation

First, the psychological specialist nurses in the team comprehensively evaluated each patient’s needs and psychological state. Psychological specialist nurses instructed patients to conduct self-assessment through questionnaires, including the Generalized Anxiety Disorder-7 questionnaire. Second, based on the results of the patient assessments and on-site interviews, appropriate intervention plans were formulated with the assistance of the psychological specialist nurses and nurse in charge. The interventions included psychological counseling and health education, guidance regarding psychological relaxation training, supervision of drug use, and observation.

##### Preoperative preparation

Prior to the operation, the team assessed the basic condition of the patient and assisted him/her so that the results of the relevant examinations could be improved. Cardiotonic, diuretic, and nourishing myocardial and other treatments were administered as directed, and the patient’s vital signs, intakes and outputs, and cardiac function were monitored daily to improve the patient’s tolerance to surgery. The responsible nurses guided the patients in their performance of the exercises designed to improve respiratory function and enhance the effectiveness of coughing (e.g., abdominal breathing, lip contraction breathing). Moreover, the team guided patients in their attempts to master ankle and knee joint movement.

#### Postoperative care

##### Close monitoring of vital signs

Patients were transferred to the ICU after the surgery for continuous electrocardiographic (ECG) monitoring, routine ventilator-assisted breathing, and regular monitoring of the results of the blood gas analyses. The responsible nurse observed closely and recorded the vital signs, including body temperature, heart rate, heart rhythm, blood pressure, respiration, and blood oxygen saturation and reported abnormalities to the doctor, timely. Endotracheal intubation was discontinued when the patient was conscious, the vital signs were stable, and hemodynamic stability was observed.

##### Pipeline care

Following the transapical mitral valve clamping, the patients underwent central venous, radial artery, and thoracic drainage catheterization. The responsible nurse monitored the patient’s central venous and radial artery punctures for blood leakages and catheter slippage. Additionally, the responsible nurse determined whether there was bleeding in the incision at the apex and whether hematoma and ecchymosis were present in the nearby skin. To avoid infection, catheters were changed once a week or in cases of bleeding, fluid leakage, edge curling, and moisture. All the catheters were placed appropriately to prevent distortion. Within 4 h after surgery, the thoracic drainage tube was squeezed every 30 min; the amount, color, and nature of the drainage fluid were observed; and the drainage tube was removed as soon as possible. The responsible nurse paid attention to changes in urine volume and character and cleaned the urethral orifice every shift. The arterial manometry tube was zero-calibrated at least once per shift and flushed with heparin at least once every 2 h to prevent tube blockage and ensure the accuracy of invasive blood pressure measurements.

##### Postoperative pain management

Pain nurses conducted pain management for patients, which involved the assessment of pain status and the effectiveness of pain relief. To assess wound pain, the pain nurse first evaluated patients using the Critical-care Pain Observation Tool (CPOT) and reported the need for analgesic treatment to the doctor if the score was more than three points. Psychological comfort was provided if the score was less than two points.

##### Postoperative nutritional support

Specialized nutrition nurses carried out nutrition support management for the patients, including nutrition risk assessment, nutrition route selection, nutrition type, calorie intake, and nutritional indicator monitoring. First, the European Nutrition Risk Screening (NRS) was used to conduct nutrition screening for each patient, and each patient participated in the formulation of their own nutrition support plan. Oral feeding was preferred. The importance of protein intake was emphasized, and thus, they followed a high-calorie, high-protein, and high-vitamin diet. Early enteral nutrition was provided to patients who could not eat by mouth. In these cases, the nurse utilized a constant-temperature infusion pump to provide nutrition, starting with a small dose and gradually increasing the amounts while monitoring for clinical manifestations of feeding intolerance. The nurse observed changes in the amount of water intake and output and in nutritional indicators (albumin and total protein).

##### Rehabilitation nursing

The rehabilitation nurse was responsible for the evaluation and guidance of the postoperative rehabilitation training. Postoperative rehabilitation treatment included mainly postural management, breathing training, and anti-venous thromboembolism (VTE) treatment. Proper postural management refers to the use of gravity to drain and discharge secretions from the lung lobes or segments. For patients with sedation and disturbance of consciousness, the bed head was raised (by 30°–45°) when the physiological condition permitted. Breathing exercises can fully expand the lungs, promote the discharge of secretions in the alveoli and airways, avoid the accumulation of sputum at the bottom of the lungs, increase lung capacity, and enhance lung function. Therefore, rehabilitation nurses guided patients to participate actively in lung physical therapy, such as abdominal breathing, lip-reducing breathing, effective coughing, turning over and patting the back, and vibrator expectoration treatment. The anti-VTE treatment included upper limb muscle strength exercises, ankle pump exercises, and hip-lifting exercises. After removal of endotracheal intubation, patients were provided immediately with muscle strength training of the upper limbs (e.g., grip strength test) and active and passive movements of the lower limbs, including ankle pumping, knee joint movements, and hip-lifting movements. After the patients were transferred to the general ward, they received bedside sitting training, bicycle training, bedside standing training, auxiliary walking training, and ward walking training.

##### Prevention of and nursing treatment for complications

Pericardial tamponade

Transapical mitral valve clamping was performed by cutting open the apex of the heart and inserting the clamp, following which the purse was sutured to stop bleeding. Due to the strong contractility of the left ventricle and fast blood flow, patients are at risk of bleeding from the apical wound after surgery. Pericardial tamponade can result if the apex ruptures and massive bleeding penetrates the pericardial dissection. In such cases, the responsible nurse is required to squeeze the drainage tube regularly; observe the amount, color, and properties of the drainage fluid; and monitor the patient’s vital signs, closely. In case of a sudden drop in blood pressure, rapid increase in heart rate, high central vein, or distant auscultation heart sound, the nurse is required to report to the doctor immediately.

ValveClamp shedding/displacement

Operation methods and cardiac hemodynamics can cause the valve clamp to fall off or shift, resulting in emboli and the aggravation of mitral regurgitation. After the operation, the responsible nurse is required to observe the patient’s vital signs and symptoms, closely. If chest tightness, dyspnea, or precordial pain occurs, the nurse is required to report to the doctor promptly.

Infection

Because of the age, the surgical patients are considered to have a weak immunity. Nurses are required to pay attention to the aseptic status during operation, body temperature, and C-reactive protein and procalcitonin levels. Once signs of infection have been identified, the nurse is required to report to the doctor, use antibiotics routinely as required, and remove all tubes timely.

##### Nursing with antiplatelet and anticoagulant drugs

The implanted mitral valve clamp is made of a cobalt chromium alloy and is thus regarded as a foreign body. Given that it may form a thrombus in the body and cause emboli, anticoagulant therapy is required after the operation [4]. The doctor determines the anticoagulation plan based on the postoperative bleeding at the incision, amount of drainage fluid, and prothrombin time. During the medication treatment period, medical workers are required to minimize invasive procedures such as punctures and extend the compression time after punctures. In this study, patients were closely monitored for bleeding conditions (e.g., gingival bleeding, petechiae, skin ecchymosis, subconjunctival bleeding, nosebleed, and melena).

##### Discharge education

The multidisciplinary collaborative nursing team developed targeted discharge education for patients that included the following advice: avoidance of heavy physical labor, properly graded increases in exercise over the 3-month period after the operation, avoidance of crowded places, and residence in a ventilated environment. Moreover, patients were instructed to stop smoking, limit alcohol use, and follow a low-salt, low-fat, easy-to-digest, and light diet. In addition, they were instructed to maintain unobstructed defecation, using a glycerine enema as appropriate. The nurses highlighted the importance of preventing colds and returning to the hospital in cases of respiratory tract infections. Patients were informed about the effects and adverse reactions of drugs and instructed to take the drugs regularly and not to increase or decrease drug dosages without authorization. The nurses further instructed patients to monitor for signs of bleeding and their blood pressure and pulse rates and urine volumes. In cases of bleeding for 3 consecutive days, a fasting weight gain of more than 2 kg, chest tightness, dyspnea, or edema, other conditions, the patients were advised to visit the hospital, promptly. Follow-up visits were conducted at 1, 3, 6, and 12 months after discharge.

### Statistical analyses

All data were analyzed using SPSS 25.0 statistical software (SPSSInc.). Normally distributed data were described as means and standard deviations, and non-normally distributed data were represented by medians and interquartile ranges (*P*25, *P*75). The count data were described using frequencies and percentages, and the Wilcoxon signed rank test was used to compare the effects before and after intervention. *P* < 0.05 was considered statistically significant.

## Results

In this study, 10 patients admitted to Fujian Heart Medical Center between June 2020 and June 2021 who underwent transapical mitral valve repair were selected; no psychopathic patients were found. Therefore, all 10 patients were included in this study. All the selected patients completed the investigation and collection of general data and clinical data.

### General information and clinical data

Our sample of 10 patients included 4 men (40%) and 6 women (60%), with a mean age of 71.4 ± 5.2 years. Among them, one patient had a history of an anterior descending branch coronary intervention, while seven had hypertension, three had diabetes, and three had chronic obstructive pulmonary disease. Transesophageal echocardiography revealed that all the patients had posterior mitral valve prolapses with severe regurgitation. The preoperative reflux grades were all severe; the mean left ventricular ejection fraction was 69.9 ± 6.4%, and the mean left atrial inner diameter and diastolic left ventricular inner diameters were 45.4 ± 4.4 cm and 56.1 ± 2.8 cm, respectively. The New York Heart Association cardiac function grade III accounted for 80% of cases, as shown in Table 1. The median Euroscore II score was 2.88% (2.35, 7.41%). All the patients underwent preoperative examinations and transapical mitral valve pinching under general anesthesia. The device performed well, no circulatory assistance was required during the operation, and no adverse events occurred. The mean operation time was 86.9 ± 43.2 min.

**Table 1:** Baseline data of patients undergoing transapical mitral valve clamping

Variable	Total (*N* = 8)	
	N	%
*Demographic factors*		
Age, years		
60 ~ 70	4	40
> 70	6	60
Mean	71.4	
SD	5.2	
Sex, male	4	40
Smoker	3	30
*Past medical history*		
Hypertension	7	70
Diabetes	3	30
Chronic obstructive pulmonary disease	3	30
Grade of mitral regurgitation		
severe	10	100
NYHA heart function grade		
Grade II	2	20
Grade III	8	80
Left ventricular ejection fraction (%)		
60 ~ 70	5	50
70 ~ 80	5	50
Left atrial diameter (mm)		
40 ~ 50	6	60
50 ~ 60	4	40
Diastolic left ventricular diameter (mm)		
50 ~ 55	6	60
56 ~ 60	4	40

*NYHA* New York Heart Association

### The efficacy of psychosocial interventions

In this study, preoperative anxiety scores were significantly higher than post-intervention scores (15.2 ± 2.3 vs. 6.7 ± 1.8, *P* < 0.05), psychological intervention measures before and after the intervention of anxiety score difference were statistically significant, and psychological interventions can reduce the anxiety level of patients before surgery, as shown in Table 2.

**Table 2:** Comparison of the anxiety levels in patients undergoing transapical mitral valve clamping before and after the psychological interventions

Variables	Before the intervention	After the intervention	*Z*	*P*
Generalized anxiety Screening Scale Score	15.2 ± 2.3	6.7 ± 1.8	−2.840*	0.005

* Wilcoxon signed rank test

### Vital signs monitoring status

In this study, the median duration of mechanical ventilation for the ten patients was 5.8 h. Abnormal postoperative vital signs were as follows: One patient experienced hypothermia (34.5 °C) after the operation and low heart rate (52 beats/min), which returned to 36.9 °C and 82 beats/min, respectively, after re-warming for 2 h. Postoperative ECG monitoring revealed the presence of premature ventricular beats in one patient. The blood gas analysis of this patient indicated a potassium concentration of 3.3 mmol/L, which was related to the patient’s long-term use of potassium-draining diuretics prior to the surgery, postoperative diuretic use, and failure to supplement with potassium appropriately. Premature ventricular beats disappeared after potassium supplementation, and the re-examination of the blood gas indicated a potassium concentration within the normal range.

### Pipeline care

In this study, no abnormalities such as wound infection or catheter slippage were observed among our ten patients during hospitalization. The median duration of indwelling for the drainage tube in this study was 2 days.

### The efficacy of postoperative pain management

In this study, four patients experienced wound pain, which was relieved after treatment with bucinnazine. In both patients, the CPOT scores were reduced from 6 points to 2 points.

### The efficacy of postoperative nutritional support

In this study, preoperative NRS scores were ≥ 3 points in six patients, 60% of whom exhibited nutritional risk. In this study, none of the patients had symptoms of feeding intolerance such as abdominal distension, diarrhea, or vomiting after eating. The mean albumin (g/L) decreased from 39.2 ± 5.2 before surgery to 33.5 ± 2.8 after surgery, while the total protein (g/L) decreased from 69.2 ± 6.4 before surgery to 63.5 ± 2.3 after surgery, as shown in Table 3.

**Table 3:** Comparison of the nutritional indexes before surgery and at discharge of patients undergoing mitral valve clamp surgery

Variables	Before the intervention	At discharge	*Z*	*P*
Albumin (g/L)	39.2 ± 5.2	33.5 ± 2.8	−2.547*	0.011
Total protein (g/L)	69.2 ± 6.4	63.5 ± 2.3	−2.448*	0.014

* Wilcoxon signed rank test

### The efficacy of rehabilitation nursing

In this study, the color ultrasound showed that mitral regurgitation of 10 patients was ≤2+ at discharge. Among the 10 patients, the Activity of Daily Living Scale scores at discharge was significantly higher than the preoperative scores (69.0 ± 4.6 vs. 55.0 ± 5.8). In addition, the 6-min walking test results at discharge were significantly better than before surgery (318.0 ± 21.7 m vs. 295.2 ± 18.4 m). As shown in Table 4, transapical mitral valve clamping reduced the degree of mitral regurgitation, increased the patients’ self-care ability and 6-min walking distance (*P* < 0.05).

**Table 4:** Comparison of the indicators of cardiac function before surgery and at discharge of patients undergoing mitral valve clamp surgery

Variables	Before the intervention	At discharge	*Z*	*P*
Degree of reflux			−3.162*	0.002
≤ 2+	0	10 (100.0)		
> 2+	10 (100.0)	0		
Activity of Daily Living Scale score	55.0 ± 5.8	69.0 ± 4.6	−2.877*	0.004
6MWT(m)	295.2 ± 18.4	318.0 ± 21.7	−2.805*	0.005

* Wilcoxon signed rank test

### Incidence of complications

None of the 10 patients in our study experienced pericardial tamponade, ValveClamp shedding/ displacement, infection or hemorrhage during hospitalization.

## Discussion

This study found that psychological interventions of MDTs can effectively reduce preoperative anxiety level in patients undergoing transapical mitral valve clamping. Zhang H et al. [9] confirmed that the multidisciplinary group psychological intervention mode for postoperative patients significantly reduced the level of postoperative pain and anxiety, which was consistent with the results of this study. The high preoperative anxiety scores of the study participants may have been related to older age patients, and the fact that transapical mitral valve clipping is a new surgical procedure. Therefore, patients had considerable concerns about whether and when to undergo the operation, the safety of the procedure, and the expected surgical outcomes. Hence, nurses cooperated with the responsible doctors in the clinical work, actively communicated with patients and their families, explained the patient’s progress, and helped patients understand the dangers of severe mitral regurgitation without surgery and the advantages of transapical mitral valve clamping. These repeated and effective explanations allowed patients and their families to develop a sense of comfort with the new technology. This reduced the anxiety of the patients and their families and increased their trust in the medical workers.

In this study, it was found that the MDTs collaborative nursing group resulted decreasing in pain scores and relief of pain. Studies have confirmed the importance of MDTs in pain management, especially pain assessment, and that the use of MDTs can improve patient pain management, pain education, outcomes, and satisfaction [10, 11]. These findings were consistent with the results of this study. Kehlet H [12] pointed out that postoperative pain can lead to the reflex contraction of abdominal and chest muscles, atrophy of alveoli, and a decrease in tide volume and alveolar ventilation, resulting in hypoxemia and a decreased oxygen supply capacity of the tissues. Postoperative pain can limit cough, resulting in poor sputum, airway obstructions, atelectasis, and secondary pulmonary infections, aggravating the degree of hypoxia. At the same time, postoperative pain can reduce the levels of immunoglobulin, reduce the immune capacity of the body, and increase the risk of postoperative infection. In addition, pain improves sympathetic nerve activity, accelerates cardiac rate, increases blood pressure, and increases peripheral vascular resistance [13]. Therefore, effective pain management can reduce the incidence of various postoperative complications [9].

This study found that 60.0% of patients with mitral valve clipping were at risk of malnutrition before surgery. A study of 712 patients undergoing cardiac surgery found that the incidence of malnutrition was 22.9%; Among them, patients with valvular disease, severe heart function transmission, renal insufficiency, and high inflammation indicators were the most prone to malnutrition [14]. Disease-related malnutrition is a serious problem for patients undergoing cardiac surgery because it not only leads to higher mortality, risk of postoperative infections, and other complications, but it also prolongs hospital stays [15, 16]. Therefore, the early identification of patients with poor nutritional status during the perioperative period is crucial to ensure optimal nutritional assessments and interventions [16]. In addition, in a study of 250 patients after cardiac surgery, it was found that the 6MWD at discharge was longer in the group with an adequate postoperative dietary intake than in the group with an inadequate postoperative dietary intake and that active nutritional interventions, early after surgery, helped to support functional recovery [17]. These findings indicated that nutritional support should be strengthened among postoperative patients by providing a high-protein and low-fat diet.

In this study, under the guidance of the nurses in the rehabilitation department in MDTs, the self-care ability and 6-min walking distance of the subjects were both increased compared with those before surgery. Rehabilitation exercise can train the muscles of the whole body, prevent muscle atrophy and common peroneal nerve damage, promote circulation, and prevent deep vein thrombosis. Early rehabilitation exercise is an important part of accelerated rehabilitation surgery. After cardiac surgery, attention to the maintenance of cardiac function is required to avoid any decompensation of cardiac function due to excessive exercise [18]. However, studies have found that it was feasible, safe, and effective to use an evaluation-based cardiac rehabilitation program to enhance the independence and mobility of patients after transcatheter aortic valve replacement [19]. A multicenter study found that early exercise training program can significantly increase left ventricular ejection fraction and activity endurance in patients undergoing transcatheter aortic valve repair [20].

The results of this current study should be interpreted considering the following limitations. First, this project was a single-center prospective study with a small sample size. A multicenter prospective cohort study with a large sample size is recommended to further verify the nursing effect of multidisciplinary collaboration group on patients undergoing transapical mitral valve clamping. Second, we only analyzed the results of hospitalized patients and did not follow up patients after discharge. Therefore, we were unable to determine the long-term impact of MDT on patients with transapical mitral valve clipping.

## Conclusion

The present report illustrates that utilizing a multidisciplinary nursing team can eliminate the barriers among different nursing specialties, allowing for cross-discipline collaboration and the implementation of complementary management strategies that promote the integration of nursing resources and improve patient care. Thus, the use of MDTs allows medical workers to provide more comprehensive and systematic care for patients undergoing a mitral valve clamping operation, thus promoting patient recovery.

## Data Availability

The datasets used and/or analyzed during the current study are available from the corresponding author on reasonable request.

## Abbreviations

ECG: Electrocardiography
CPOT: Care Pain Observation Tool
ICU: Intensive care unit
MDT: Multidisciplinary team
NRS: Nutrition Risk Screening
VTE: Venous thromboembolism
6MWT: 6-min walking test
